# Insurance Type Influences Access to Biologics and Healthcare Utilization in Pediatric Crohn’s Disease

**DOI:** 10.1093/crocol/otab057

**Published:** 2021-08-07

**Authors:** Jose Antonio Quiros, Annie Lintzenich Andrews, Daniel Brinton, Kit Simpson, Annie Simpson

**Affiliations:** 1 MUSC Children’s Hospital, Division of Pediatric Gastroenterology, Hepatology and Nutrition, Charleston, South Carolina, USA; 2 MUSC Children’s Hospital, Department of Pediatrics, Charleston, South Carolina, USA; 3 MUSC College of Health Professions, Department of Healthcare Leadership and Management, Charleston, South Carolina, USA

**Keywords:** Crohn’s disease, healthcare access, biologic utilization, pediatric

## Abstract

**Background:**

The objective of this study is to determine if there is an association between insurance status and access to biologics among children with Crohn’s disease (CD). Additionally, we seek to determine differences in healthcare utilization between these groups, utilizing a national sample of children with CD.

**Methods:**

Children aged 8–18 with a diagnosis of CD were identified from 2012–2016 Truven Health MarketScan (IBM Watson Health). Patients were classified into Public/Medicaid or as Commercial/Privately Insured. Descriptive statistics were compared between groups and sensitivity analysis performed using inverse probability of treatment weighting. Adjusted differences in healthcare utilization were estimated by multiple linear regression models.

**Results:**

We identified 6163 patients with a diagnosis of CD. There were no significant differences in each payer group’s demographic characteristics, comorbidities, or surgery rates. Over the 18-month follow-up period, 132 (20.4%) subjects in the public insurance group and 851 (15.4%) children in the private insurance group received biologics. Medicaid patients were 39% more likely to receive a biologic agent within 18 months of diagnosis compared to privately insured children (*P* = .0004). Postdiagnosis rates of hospitalizations and Emergency Department visits were significantly higher for the Medicaid group.

**Conclusions:**

In this national sample of children with CD, publicly insured children were more likely to receive a biologic within 18 months of diagnosis compared to children with private insurance. At all points in time, publicly insured children also utilized emergency room services and required hospitalization at a significantly higher rate.

## Introduction

The development of therapeutic biologics has been an important advancement in the management of autoimmune disorders and cancer.^1^ Biologic agents are made through recombinant genetic technology instructing laboratory “host” cells to create specific proteins, then repurposed for use in humans.^1^ These compounds are changing how we manage different healthcare conditions but patient access to these potentially life-saving drugs is limited due to high cost.^2^ The cost of these drugs can be up to several thousand per dose. Recent economic estimates expect revenue from biologics to reach $390 billion worldwide for 2025.^3^ The entry of generic copies of some of the older biologics or biosimilars has opened the door to lower costs but the issue of access remains as insurance providers limit approval to contain healthcare costs of their covered population citation.^4^

Biologic agents that target tumor necrosis factor-a (anti-TNF) entered the health market in 1998. The indications for these drugs have expanded over time to include psoriasis, rheumatoid arthritis, and inflammatory bowel disease (IBD) (Crohn’s disease [CD] and ulcerative colitis). Longitudinal experience with the use of biologics in pediatric patients with IBD support the earlier use of these agents with improved mucosal healing rates and lower risk of fistula formation.^5,6^ The cost of these drugs varies with average costs per-infusion of $1974 (USD) for the most common intravenous formulation (infliximab).^7^ It is estimated that the average yearly cost of biologics in adults with IBD is $17 000 per person in the first year of therapy.^7^ As of January 2020, there are 12 biologics approved for IBD use in adults, 9 of which target TNF, and 11 new biologics in different stages of development targeting 9 new molecular markers.^4^ With an ever more dense landscape, understanding impact of insurance on medication access and how this might affect healthcare utilization is paramount.

The annual aggregate economic burden of CD in the United States was 6.3 billion (USD) in 2013.^8^ Earlier treatment of CD with anti-TNF has been suggested as the best treatment approach, with better clinical response and mucosal healing noted.^9^ This was also found in children with CD, with larger outcome effect than those noted in initial trials in adults.^10,11^ These efficacy data have helped increase the use of biologics to about 15%–20% of all adult CD patients on some biologic drug in the United States and Europe.^12,13^ Prescription associated costs have also increased in this patient population. Currently, in the United States and Europe, prescriptions account for about 50% of the direct disease-related economic burden of CD.^8,12^ In the United States, the Centers for Medicare and Medicaid Services (CMS) has tried to streamline guidelines for biologic application with some success, while private insurance companies and health management organizations have tried to control their costs through a structured and rigid preauthorization process with mixed results.^14,15^

The objective of this study is to determine whether the current differential drug approval methods between privately (commercial) and publicly insured children is associated with access to biologics in a national sample of children with CD. We will also examine if there are any healthcare utilization differences associated with insurance type. Patients with Medicaid fee-for-service, insurance coverage for any FDA approved medication is guaranteed if appropriately prescribed by a physician.^16^ How clinical characteristics affect these outcomes will be examined as part of the analysis. We hypothesize that children with Medicaid will have a shorter interval between diagnosis and start of biologic therapy compared with privately insured children in the first 18 months after diagnosis of CD. Whether other factors, like demographics or disease characteristics, might impact this outcome will be determined in this population.

## Materials and Methods

2012–2016 Truven Health MarketScan Medicaid and Commercial Insurance databases were used for this analysis. Only patients who had complete data were included in analysis and Managed Medicaid products were not included as they often are managed by private for-profit payers and thus not appropriate as a comparator in this case. Patients had to be continuously enrolled during the study period (6-month preindex and 18-month postindex). We identified children aged 8–18 with the diagnosis of CD using ICD-9 codes 555.XX and ICD-10 codes K50.XX (Supplementary Table). Patients under 8 years of age at diagnosis were excluded from the analysis to avoid including very early onset IBD patients which are perceived to be a clinically distinct population.^17^ Children were classified by payer type into 2 categories: Public/Medicaid fee-for-service and Commercial (private) Insurance (PI). Demographic characteristics included sex, age, and race (not reported for commercial insurance patients). Date of CD diagnosis was defined as the endoscopy date on or before the first incidence of a CD diagnosis code in the patient’s record, and is referred to herein as the index date. Healthcare utilization and patent clinical characteristics were examined for the 6-month preindex period for comorbidity assessment. Outcomes including receipt of biologic, time to first biologic, and Emergency Department (ED) or hospitalization rates which were examined during an 18-month postindex period. Patient disease associated risk factors examined included: abdominal pain, growth disturbances (failure-to-thrive, growth failure, and short stature), recurrent or persistent fever, presence of internal and external fistulas, joint disease (arthritis), skin manifestations (erythema nodosum, pyoderma gangrenosum), fever, and eye disease (uveitis) were evaluated (see Table 1). Surgical complications including bowel resection, ostomy creation, and colectomies were identified before and after diagnosis in both groups.

**Table 1.** Clinical characteristics preindex date

	*N* (%)	Private insurance (%)	Medicaid (%)	*P*
Total number (*N*)	6163	5518	645	
Characteristic				
Sex (M)	3436 (55.7)	3070 (55.6)	366 (56.7)	.5
Age, mean (years) [median/SD]		14.8 [15/2.7]	14.6 [15/2.8]	.2
Abdominal pain	3106 (50.4)	2735 (49.6)	371 (57.5)	.0001
Gastroesophageal reflux	609 (9.8)	518 (9.4)	91 (14.1)	.0001
Growth failure	369 (5.9)	328 (5.9)	41 (6.4)	.6
Malnutrition	198 (3.2)	193 (3.5)	5 (0.8)	.0002
Low weight for age	1068 (17.3)	950 (17.2)	118 (18.3)	.5
Failure-to-thrive	217 (3.5)	194 (3.5)	23 (3.6)	.9
Short stature	221 (3.6)	202 (3.7)	19 (2.9)	.3
Fever	544 (8.8)	486 (8.8)	58 (9.0)	.8
Anal fissure/fistula	306 (4.9)	264 (4.8)	42 (6.5)	.05
Arthritis	61 (1.0)	50 (0.9)	11 (1.7)	.05
Appendicitis	50 (0.8)	50 (0.9)	0 (0)	.015
Erythema nodosum	43 (0.7)	40 (0.7)	3 (0.5)	.4
Uveitis	5 (0.08)	4 (0.1)	1 (0.2)	.5
*Clostridium difficile* colitis	117 (1.9)	109 (2.0)	8 (1.2)	.2

### Statistical Analysis

The primary outcome variable was number of days from index date to date of first biologic claim. For patients not receiving a biologic agent, time was censored at the last day of the study. Secondary healthcare utilization outcomes included number of ED visits or hospital admissions within 18 months from the index date. Descriptive statistics were compared between groups using chi-squared tests for categorical variables and 2-sample Student’s *t*-test for continuous variables (or nonparametric Wilcoxon scores if appropriate). Kaplan–Meier curves were used to estimate and visualize unadjusted time to first biologic by insurance group with *P* values reported for the log-rank statistic. Cox proportional hazards regression was used to estimate adjusted hazard ratios between the comparison groups while controlling for baseline covariates. Adjusted differences in healthcare utilization were estimated using multiple logistic regression. In an effort to assess if the few unequal preindex patient characteristics posed a significant selection bias threat to study findings, a sensitivity analysis was performed using inverse probability of treatment weighting (propensity score weighting) including all demographic and clinical characteristics described during the baseline 6-month preindex period. Sensitivity analysis using propensity score indicated no selection bias effect on adjusted study findings, thus primary results are reported using adjusted models without propensity scoring. SAS version 9.4 (SAS Institute Inc.) was used for analysis. All tests were 2 sided and statistical significance tested at α < .05.

## Results

**Figure 1.**
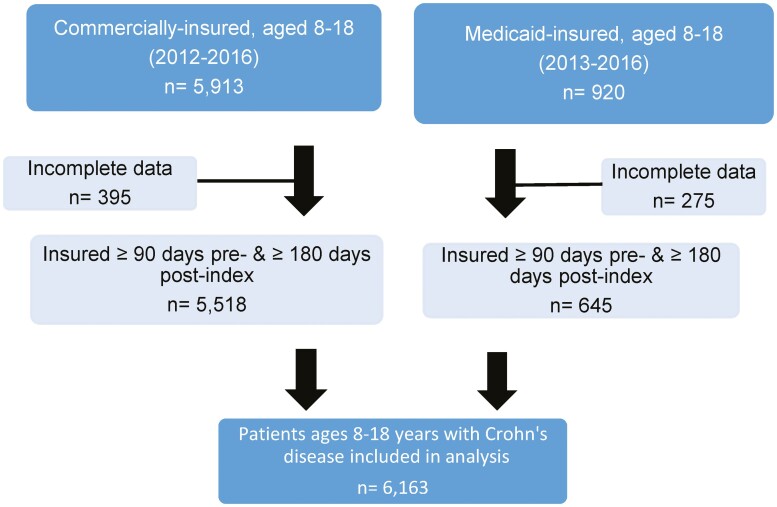
Consort diagram.

We identified a total of 6163 children with CD, of these 5518 were privately insured children and 645 children had Medicaid fee-for-service and had all necessary data points to perform this analysis (Figure 1). There were no significant differences between each group’s baseline characteristics such as age, sex, and incidence of comorbidities (Table 1). In the 6-month preindex period, Medicaid patients had a higher proportion of anal fissure diagnosis (6.5% vs 4.8%, *P* value = .05). At initial endoscopy/index date, patients in the Medicaid group had a higher proportion of having a perianal fistula (1.1% vs 0.4%, *P* = .05) while patients with private insurance had a higher incidence of diagnosed growth failure and malnutrition (3.5% vs 0.8%, *P* value = .0002).

**Figure 2.**
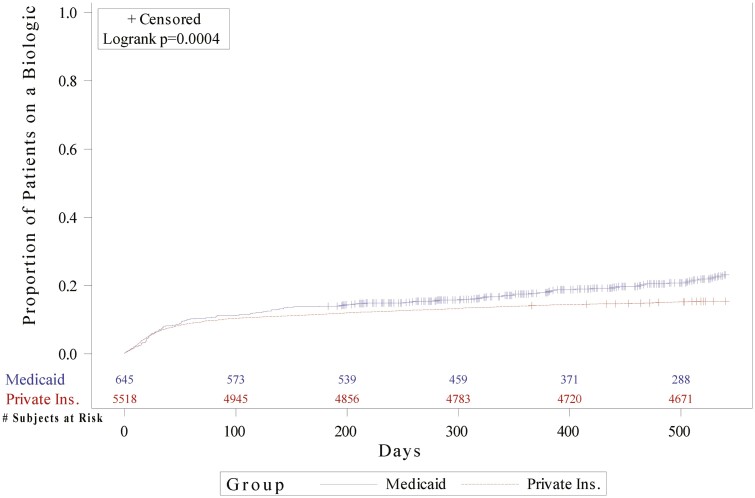
KM curve on time to biologic by insurance type over 18-month postindex period. Abbreviation: KM, Kaplan–Meier.

There was significantly higher proportion of Medicaid patients who received biologics. Over the 18-month follow-up period, 132 (20.4%) subjects in the Medicaid and 851 (15.4%) children in the PI group received biologics. Children in the PI group who went on to biologics did so sooner than children under Medicaid. Among the children who received a biologic, the unadjusted time to first biologic was 151 days in the Medicaid group vs 112 days in the PI group. Compared with the PI group, the adjusted hazard of a child covered by Medicaid receiving a biologic at any time within 18 months of initial diagnosis was 39% higher (hazard ratio 1.39, confidence interval [CI] 1.16–1.68, *P* value = .004) (Figure 2). The presence of documented arthritis and time after index date increased likelihood of biologic use in children with CD.

The odds ratio (OR) of a hospital admission was 2.27-fold higher for children who required a biologic agent (OR 2.27; CI 1.94–2.66, *P* value <.0001). While controlling for all other covariates, being on Medicaid alone yielded a 79% higher adjusted odds for hospital admission (OR 1.79; CI 1.48–2.17, *P* value <.0001) (Table 2). The odds of being hospitalized were found to increase by the presence of documented abdominal pain (OR 1.72; CI 1.51–1.96, *P* < .0001) and infection with *Clostridium difficile* irrespective of other clinical characteristics (OR 2.56; CI 1.74–3.76, *P* < .0001) (Table 2).

**Table 2.** Healthcare utilization postindex by insurance payer type

	Hospitalizations (*N* = 1306)			Emergency room visit (*N* = 2155)		
	OR	95% CI	*P*	OR	95% CI	*P*
Age	0.99	0.9–1.02	.7	1.02	1.0–1.04	.03
Male	1.18	1.04–1.32	.01	1.22	1.1–1.37	.0002
Medicaid	1.79	1.48–2.17	<.0001	2.77	2.33–3.3	<.0001
Biologic use	2.27	1.94–2.66	<.0001	1.54	1.33–1.78	<.0001
Arthritis	8.5	1.8–38.9	.005	2.67	0.6–11.78	.19
Fistula	1.04	0.78–1.38	.7	1.16	0.9–1.49	.22
Abdominal pain	1.72	1.51–1.96	<.0001	1.74	1.56–1.95	<.0001
*Clostridium difficile* infection	2.56	1.74–3.76	<.0001	1.93	1.32–2.82	.0007
Small bowel Crohn’s disease	1.08	0.83–1.41	.5	0.94	0.7–1.17	.5
Gastroesophageal reflux	1.12	0.92–1.37	.2	1.45	1.22–1.73	<.0001
Low weight	1.14	0.97–1.35	.1	0.9	0.77–1.04	.1

Abbreviations: CI, confidence interval; OR, odds ratio.

The odds of ED visits were increased in Medicaid patients by 2.77-fold (OR 2.77; CI 2.33–3.30, *P* < .0001). Children on biologics had a 54% higher odds of ED visits (OR 1.54; CI 1.33–1.78, *P* < .0001) (Table 2). Emergency use had other significant variables with younger children and males having a higher odds of going to the ED (*P* value .03 and .0002, respectively). Similar to hospitalization trends, the risk of ED use was higher in patients with abdominal pain and *C. difficile* infections (*P* value <.0001 and .0007, respectively) (Table 2). What was surprising was the strong association of reported gastroesophageal reflux with ED visits (OR 1.45; CI 1.22–1.73, *P* value <.0001). Overall, rates of major complications requiring surgery like colon resection, need for an ostomy and bowel resection were similar in both the PI and Medicaid groups (Table 3). Children in the PI group received parenteral nutrition at a higher rate than children covered under Medicaid (*P* value .04), impact of insurance on this decision making paradigm was unclear from the data. Insurance type did influence the number of ED visits per subject with Medicaid patients having 0.5 ED visits per subject compared to 0.3 visits for PI patients (*P* < .0001).

**Table 3.** Postindex outcomes by insurance type

	Commercial insurance	Medicaid	*P*
*N*	5518	645	
Any biologic, *N* (%)	851 (15.4)	132 (20.5)	.004
Adalimumab, *N* (%)	777 (14.1)	113 (17.5)	—
Infliximab, *N* (%)	74 (1.3)	19 (2.9)	—
Outcomes			
Bowel resection, *N* (%)	201 (3.6)	20 (3.1)	.4
Colectomy, *N* (%)	30 (0.5)	4 (0.6)	.8
Colostomy, *N* (%)	12 (0.2)	1 (0.2)	.7
Parenteral nutrition, *N* (%)	91 (1.6)	4 (0.6)	.04
ED visits per subject, mean (SD)	0.8 (2.2)	1.8 (3.6)	<.0001
Hospital admissions per subject, mean (SD)	0.3 (1.0)	0.5 (1.1)	.3

Abbreviation: ED, Emergency Department.

## Discussion

The advent of biologic therapy has changed long-term management of many autoimmune disorders and these drugs are being used in children more regularly. This study aimed to determine if there was any impact of payer type on 2 critical factors affecting biologic use and ultimately cost in children with CD care. First, timely access to an anti-TNF antibody therapy (biologic agents) for children with CD. Second, impact of insurance type on resource utilization. In this sample of 6163 children followed 18 months from initial diagnosis (diagnostic endoscopy date), children with Medicaid fee-for-service insurance had a higher chance of starting a biologic agent compared to children covered by PI plans. They also had higher rates of ED use and hospital admissions. Among those who received a biologic, the time to start of therapy was shorter for PI patients yet overall Medicaid patients went on a biologic at a higher rate in 18 months after diagnosis. Earlier start of biologic therapy has been associated with better outcomes in CD so early access to therapy could be thought to convey an advantage in this setting.^9^

In a recent cost analysis undertaken by the Crohn’s and Colitis Foundation, ED use was associated with an increase of healthcare associated costs in IBD.^18^ Healthcare costs in patients under 25 years of age were also noted to be higher which matches Canadian data also published in 2020.^18,19^ In the Canadian cohort, they looked at all subjects living in the Manitoba region excluding only military staff, police officers, and prison inmates. In this population, they noted that the inpatient care cost from CD was tending down since biologics were introduced.^19^ This downward trend did not extend to patients in the <25 years age group who are admitted at much higher rate and have larger yearly disease attributed costs, $12 677 vs $7324 (2015 adjusted Canadian dollars).^19^ Specific drivers of increased healthcare use in this population were not addressed in any of these studies as we have done here.

Preindex clinical characteristics were overall similar for both groups. Reported arthritis was not surprisingly associated with higher rate of biologic use in 18-month postindex date. Patients on Medicaid had a significantly higher reported rate of perianal disease like fissure or fistulas (6.5% vs 4.8%, respectively, *P* = .05). This might be a valid reason for patient moving to a biologic agent within the first 18 months after diagnosis. On the other hand, diagnosis of malnutrition was higher in the PI group (3.5% vs 0.8%, respectively, *P* = .0002). This likely represents better preventive care services, yet these patients did not go on biologics at a higher rate in this study group in spite of this notable morbid condition. Other clinical characteristics were not different between groups and did not account for the variability of biologic use noted (Table 1).

Payer type seemed to have an impact on ED use and hospital admissions. Analysis of preindex healthcare utilization showed higher rates of ED care and hospitalizations for patient covered under Medicaid. These data are not surprising and matches what has been observed by other investigators in other clinical settings.^20^ Postindex, patients in the Medicaid group had a much higher rate of hospitalization and ED visits compared to the PI group. The risk of hospitalization was higher in patients with *C. difficile* colitis in the postindex period. This matches data previously reported by us for children with IBD in South Carolina and, in that study, infection was noted to be a strong driver of healthcare associated cost in IBD.^21^ A recently published systematic review of costs in children with IBD, suggested an increase in cost of hospital admissions for pediatric patients with CD.^22^ We will note that this systematic review includes some of our previously published data as a reference. In our study, rates of surgical complications (colectomy rate, intestinal resection, and colostomy) were comparable between both groups postindex (Table 3). The presence of comorbid conditions, in the preindex and at diagnosis, was no different between payer types. Because of the similarity of the patient distribution during the baseline period, a sensitivity analysis using propensity score methods yielded the same results. The presence of arthritis was a notable driver of hospitalizations for the whole population which is not surprising but not ED use (Table 2).

In our study, we saw a true discrepancy in access to therapy by insurance type in children with IBD. Clinical characteristics being equal, there is no clear explanation for this phenomenon. A recent review of medication access in cancer patients showed a similar phenomenon in access to therapy after Affordable Care Act (ACA) expansion increased Medicaid coverage for vulnerable individuals.^23^ In this study, the time to treatment initiation was shorter in Medicaid patients even though diagnosis rates where the same for both Medicaid and commercially insured individuals. The associated commentary noted that the delay in treatment is likely due to increased administrative obligations such a prior authorization leading to “longer wait times.” ^24^

As we saw in our results, rates of admissions and ED visits were higher for Medicaid patients compared to PI patients and thus expected costs would be expected to be higher for Medicaid patients as suggested in the Park et al study. This warrants further analysis and we suspect it will highlight health access disparities in basic preventive care forcing patients to seek acute care in a hospital setting and possibly highlight need for active health “navigation” to lower healthcare utilization for children with IBD. Colvin et al noted that children covered by Medicaid convey a financial challenge to free-standing children hospitals so developing strategies to resolve these issues becomes paramount.^25^

Clinical data contained in these databases have some limitations as it is dependent on adequate coding by clinical providers which can be a challenge in most scenarios.^26^ The reliability of these databases is based on the randomness of these omissions and thus the assumption that it will equal across a populations being compared.^27^ The lack of socioeconomic data like race/ethnicity, some inpatient delivered care, and household income are also limitations when using these type of data sets if we are trying to determine health disparities in a population. In this case, the lack of variability in patient morbidity suggests that, overall, the populations are balanced and outcomes comparable but healthcare utilization (hospitalizations and ED visits) was indeed higher in the Medicaid group at all points. This has significant implications and developing strategies to address this in the future could be important. In this study, there was no significant difference in the detection of extraintestinal manifestations of CD like erythema nodosum, arthritis, fever, and uveitis between payer groups (Table 1). Nutrition outcomes like low weight for age and short stature were also not significantly different between payer groups but we did see a higher rate of recorded “malnutrition” among PI patients (Table 1). This could be more a reflection of adequate outpatient care, access to a pediatrician who would submit this diagnosis.

In their review of the Kids’ inpatient database (AHRQ), academic free-standing children hospitals had a higher rate of admission for Medicaid covered patients with losses from inpatient care, several fold higher when compared to nonacademic institutions and pediatric units within larger hospital units. This despite disproportionate share payments (DSPs) which were set to phase out with enactment of the ACA.^25^ The loss of these DSP poses a significant threat to the stability of academic free-standing children’s hospitals as it does other facilities that care for a large proportion of Medicaid covered patients.^28^ This and other cost-of-care issues will be matter for further investigation and research in this area is sorely needed as we move through uncertain times in the American healthcare landscape.

## Supplementary Material

otab057_suppl_Supplementary_Materials

## Data Availability

All data here noted were derived from the MarketScan Commercial Database and MarketScan Multi-State Medicaid Database. No new data were created and data analysis details provided in Supplementary Table. Data available through MarketScan (IBM-Watson, New York, NY).
