# Supplementary material for: Insurance Type Influences Access to Biologics and Healthcare Utilization in Pediatric Crohn’s Disease
Source: Crohns Colitis 360. 2021 Aug 7;3(3):otab057. doi: 10.1093/crocol/otab057 (PMC9802312; doi:10.1093/crocol/otab057)
Supplement: otab057_suppl_Supplementary_Materials [file otab057_suppl_supplementary_materials.pdf]

Supplemental Table: Data collection parameters

| **Pre-period** | | | | |
|---|---|---|---|---|
| | *Codes* | | | |
| *Disease/condition/surgery* | *ICD-9* | *ICD-10* | *CPT* | *Comments* |
| short stature | 783.43 | R62.52 | | |
| failure to thrive (FTT) | 783.4 783.41 | R62.51 E30.0 | | FTT and delayed puberty |
| growth failure | 782.4 | R62 R62.51 | | |
| low weight | 783.2 783.22 | R63.4 | | |
| acute gastroenteritis | 558.9 | K52.9 | | |
| C difficle colitis (infection) | 8.45 | A04.7 A04.71 | | |
| anal fistula | 565.1 | K60.1 K61.0 | | |
| anal fissure | 565 | K60.1 | | |
| appendicitis | 540.9 | K35.8 | | |
| erythema nodosum | 695.2 | L52 | | |
| fever of unknown origin | 780.6 | R50.1 R50.9 | | |
| arthritis | 714 | M19.9 | | |
| uveitis | 364.3 | H20.9 | | |
| Hematochezia | 578.1 | K92.1 | | |
| Abdominal pain | 789 | R10.XX | | |
| Gastroesophageal reflux | 530.81 | K21.XX | | |
| | | | | |
| **Disease severity** | | | | |
| | *Codes* | | | |
| *Disease/condition/surgery* | *ICD-9* | *ICD-10* | *CPT* | *Comments* |
| Fistula internal | 569.5 569.81 | K63.2 K50.014 K50.114 | | |
| fistula perianal | 565.1 | K60.1 K61.0 | | |
| growth failure | 783.4 | R62 R62.51 | | |
| FTT | 783.41 | R62.51 E30.0 | | |
| intestinal obstruction | 560.0-.9 | K50.812 K50.012 | | |
| small bowel and colon dx | 555.2 | K50.80 K50.90 | | |
| arthritis | 714 | M19.90 | | |
| erythema nodosum | 695.2 | L52 | | |
| | | | | |
| | | | | |
| | | | | |
| **Outcomes** | | | | |
| | *Codes* | | | |
| *Disease/condition/surgery* | *ICD-9* | *ICD-10* | *CPT* | *Comments* |

| | | | | |
|---|---|---|---|---|
| bowel resection lap | | | 44160<br>44140<br>44141<br>44205<br>44208<br>44210<br>44212<br>44120<br>44140<br>44615 | |
| parenteral nutrition | v58.69<br>99.15 | | S9364<br>S9365<br>S9366<br>S9366<br>S9367<br>S9368 | |
| hospital admission | | | 99221<br>99222<br>99223<br>99238<br>99239 | admit and day admit |
| total cost | | | | |
| drug costs | | | | |
| ED visit | | | 99281<br>99282<br>99283<br>99284<br>99285 | |
| colostomy | | | 44187 | |
| colectomy | | | 44204 | |
